# Leishmaniasis Recidiva Cutis

**DOI:** 10.4269/ajtmh.16-0459

**Published:** 2016-12-07

**Authors:** Álvaro A. Faccini-Martínez, Aloísio Falqueto

**Affiliations:** 1 Programa de Pós-Graduação em Doenças Infecciosas, Centro de Ciências da Saúde, Universidade Federal do Espírito Santo, Vitória, Brazil

**Figure 1.**
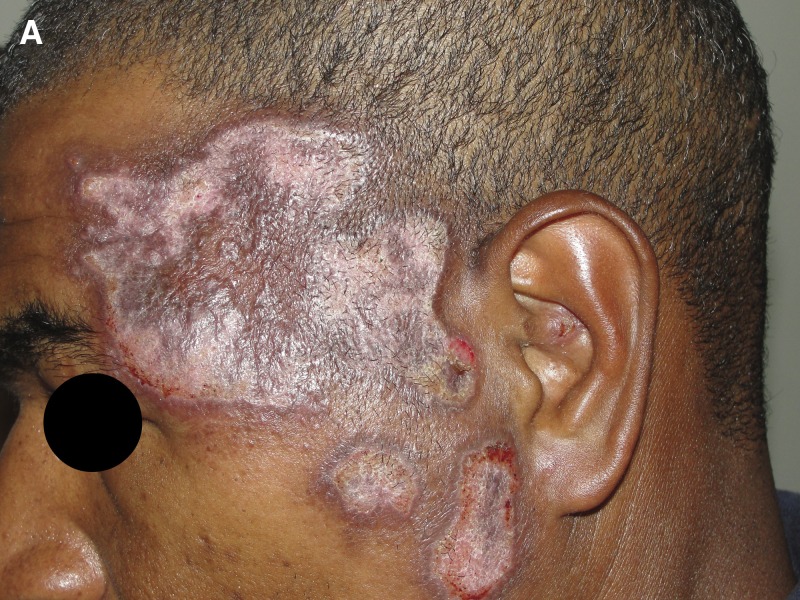
Infiltrative plaques with a central atrophic area in his left temporomandibular region.

**Figure 2.**
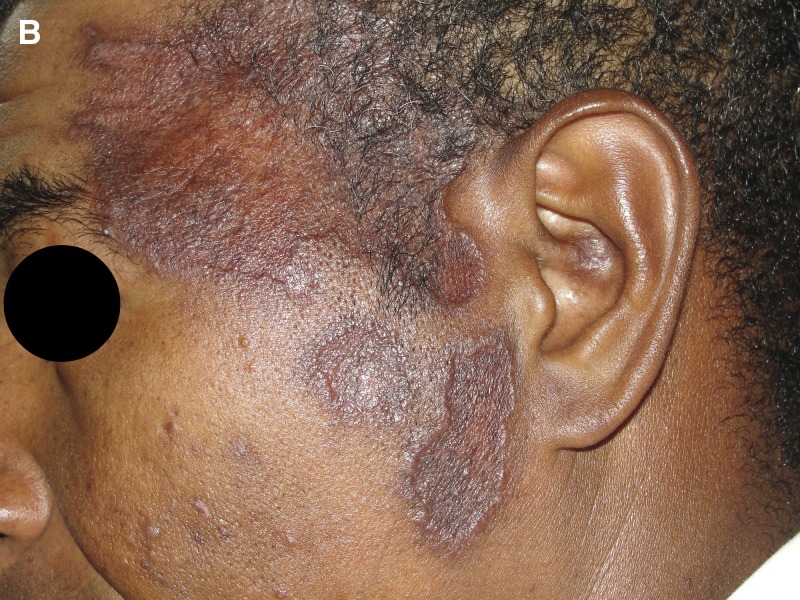
Marked clinical improvement after treatment with intravenous meglumine antimoniate.

A 39-year-old man from a rural area of Brazil (Linhares town, Espiríto Santo State) was referred to our institution for an 8-month history of pruritic lesions on the left side of his face. The lesions started as a single erythematous papule, which progressively grew, became ulcerated and associated with satellite lesions. The patient reported no local purulent discharge, no fever, or other systemic symptoms. One month before referral, he received, without clinical improvement, several courses of empirical antibiotics (gentamicin, azithromycin), topical corticosteroids (betamethasone), and drugs for leprosy (rifampin, dapsone, clofazimine). He had an unremarkable medical history. Physical examination revealed infiltrative plaques with a central atrophic area in his left temporomandibular region (Figure 1

) and ipsilateral cervical and occipital lymph nodes measuring 1 by 2 cm. Tests for human immunodeficiency virus type 1/2 (HIV 1/2 rapid test; Alere^™^, Belo Horizonte, MG, Brazil), syphilis (syphilis ultra rapid test; Alere^™^), and viral hepatitis (surface antigen of the hepatitis B virus [HBsAg] [VIKIA^®^ HBsAg rapid test; Biomérieux, Jacarepaguá, RJ, Brazil], anti-hepatitis C virus (HCV) [ImmunoComb^®^ HCV; Alere™]) were negative. The results of routine laboratory testing, including a complete blood count and a metabolic panel with liver function tests, were normal. Antinuclear antibodies were positive (1/80). Histologic examination of the skin lesions revealed an epidermis with ulceration and pseudoepitheliomatous hyperplasia, an accentuated lymphohistiocytic inflammatory infiltrate with numerous plasma cells and eosinophils, superficial and deep perivascular and interstitial distribution in the superficial dermis. An intradermal leishmanin test (*Leishmania amazonensis* MHOM/BR/73/M2269 strain) showed 11 mm of induration (read at 48 hours). A diagnosis of leishmaniasis recidiva cutis (LRC) was made. The patient was treated with intravenous meglumine antimoniate (Glucantime^®^; Sanofi Aventis, Buenos Aires, Argentina) (15 mg Sb(V)/kg/day for 20 days) resulting in a marked clinical improvement (Figure 2

). The patient remained in the improved state on the medical follow-up.

Cutaneous leishmaniasis is a vector-borne tropical infection of high prevalence in Latin America, mainly caused by the parasites *Leishmania* (*Viannia*) *braziliensis*, *Leishmania* (*Viannia*) *amazonensis*, *Leishmania* (*Viannia*) *panamensis*, and *Leishmania* (*Viannia*) *guyanensis*; the former is the predominant species in Brazil, and the only detected species in Espiríto Santo State.^1^,^2^ Localized cutaneous leishmaniasis (LCL) is the most common clinical form. The disease is characterized by ulcers which commonly start as a nodule at the site of the sandfly vector's bite, the nodule becoming an ulcer with an indurated raised outer border. LCL lesions vary in severity, clinical appearance, and time to cure.^3^ LRC is an infrequent variant of cutaneous leishmaniasis; histologically, diffuse infiltration of lymphocytes, plasma cell, and macrophages are observed, but the parasites are scarce or absent.^4^ At the same time, LRC is comparatively rare in Latin America (approximately 34 cases have been reported in Brazil in the period of 1993–2011),^1^,^4^,^5^ and such unusual clinical forms of leishmaniasis tend to be more difficult to diagnose and treat.^6^
